# Improved SERS Performance and Catalytic Activity of Dendritic Au/Ag Bimetallic Nanostructures Based on Ag Dendrites

**DOI:** 10.1186/s11671-020-03347-4

**Published:** 2020-05-24

**Authors:** Zi-Qiang Cheng, Zhi-Wen Li, Rui Yao, Kuang-Wei Xiong, Guang-Ling Cheng, Yan-Hong Zhou, Xin Luo, Zhi-Min Liu

**Affiliations:** 1 Department of Applied Physics, School of Science, East China Jiaotong University, Nanchang, 330013 People’s Republic of China; 2 Materials Interfaces Center, Shenzhen Institutes of Advanced Technology, Chinese Academy of Sciences, Shenzhen, 518055 People’s Republic of China

**Keywords:** Dendritic Au/Ag bimetallic nanostructures, Surface plasmon, Surface-enhanced Raman scattering, Catalysis

## Abstract

Bimetallic nanomaterials, which exhibit a combination of the properties associated with two different metals, have enabled innovative applications in nanoscience and nanotechnology. Here, we introduce the fabrication of dendritic Au/Ag bimetallic nanostructures for surface-enhanced Raman scattering (SERS) and catalytic applications. The dendritic Au/Ag bimetallic nanostructures were prepared by combining the electrochemical deposition and replacement reaction. The formation of Au nanoparticle shell on the surface of Ag dendrites greatly improves the stability of dendritic nanostructures, followed by a significant SERS enhancement. In addition, these dendritic Au/Ag bimetallic nanostructures are extremely efficient in degrading 4-nitrophenol (4-NP) compared with the initial dendritic Ag nanostructures. These experimental results indicate the great potential of the dendritic Au/Ag bimetallic nanostructures for the development of excellent SERS substrate and highly efficient catalysts.

## Introduction

Synergy of two or more metal materials enables the fabrication of all-in-one nanostructures with multi-functionalities [1, 2]. For instance, bimetallic nanostructures comprised of noble metals (such as Au, Ag, Pt, and Pd) exhibit special optical, electronic, and catalytic properties owing to the synergistic effect of monometallic nanostructures [1–7], and have potential applications in the fields of catalysis [5–12], surface-enhanced Raman scattering (SERS) [13–18], and sensors [19]. Nanostructured Ag are better candidates for SERS because of their low damping rate compared with Au [13, 14], yet Ag suffers from low chemical stability (e.g., surface oxidation) which limits the development of Ag-based SERS substrates with long operating lifetimes. Recently, Au/Ag bimetallic nanostructures, which sum the merits of both Au chemical stability properties and Ag strong plasmon properties, have been extensively investigated with considerably improved SERS activity and time stability [13–16].

Metal nanostructure-based catalyst with high activity and selectivity are highly desirable for chemical reactions in industry. The catalytic properties and stability of metal catalysts are generally intensified with the incorporation of the second elements [7–12]. For instance, diverse types of Au/Ag bimetallic nanostructures, such as Au-Ag hollow nanoparticles, nanowires, and nanodendrites, have been reported to exhibit superior catalytic activities to both Au and Ag monometallic counterparts [9–11]. Bimetallic nanostructures offer a promising strategy for controlled catalyzing, which could be in-suit monitored by the SERS signals in real-time [20, 21].

Branched nanostructures feature many multi-level branching nanostructures that allows abundant inter-branch gaps/junctions, edges, corners, and large surface-to-volume ratio, all of which can avail surface-sensitive applications such as localized surface plasmon resonance (LSPR), SERS, and catalysis [22–30]. Therefore, the branched nanostructure is a suitable bifunctional substrate with both plasmonic/SERS and catalytic activity. Recently, dendritic Au/Ag bimetal nanostructures have been reported. Most reports focused on the SERS activity of dendritic Au/Ag bimetallic nanostructures [15–18], but its catalytic activity was seldom explored [11]. In this work, we prepared the dendritic Au/Ag bimetallic nanostructure by combining the electrochemical deposition and replacement reaction. The plasmon properties, SERS enhancement and time stability, and catalytic activity of the dendritic Au/Ag bimetallic nanostructures were comprehensively investigated. By adjusting the replacement reaction times (morphology and composition), a tunable LSPR, excellent SERS characteristics, and high catalytic activity were obtained. Our experimental results demonstrate that dendritic Au/Ag bimetallic nanostructures can be a promising candidate for excellent SERS substrate and highly efficient catalysts.

## Experimental Methods

The dendritic Ag nanostructures were prepared through an electrochemical deposition procedure described in our previous studies [22, 23, 30]. The indium tin oxide (ITO) glass (1.5 cm × 1 cm, 17 Ω/square) and platinum (Pt) plate were used as the cathode and anode, respectively. Electrochemical deposition was then carried out in an electrolyte containing AgNO_3_ (2 g/L) and citric acid (40 g/L) at a constant current density of 1 mA•cm^–2^ for 180 s. Then, the dendritic Au/Ag bimetallic nanostructures were prepared by immersing the Ag dendrites electrodeposited on the ITO glass into 20 mL of 5 mM HAuCl_4_ solution for the replacement reaction. The samples prepared in each step were rinsed with ultrapure water to remove the residual solution and then dried under a N_2_ atmosphere. The SERS samples were prepared by immersing the dendritic nanostructures samples in 10^−9^ M 1,4-benzenedithiol (1,4-BDT) ethanol solution for 4 h. The catalytic reaction was carried out by adding a piece of catalyst (the obtained dendritic nanostructures sample) with the size of 5 × 10 mm^2^ to a mixed aqueous solution of 4-NP (1 mL, 2 × 10^−5^ M) and ice NaBH_4_ (1 mL, 6 × 10^−2^ M).

The structure and composition of the samples were characterized by using a scanning electron microscope (SEM, S4800) equipped with an energy-dispersive X-ray spectrometer (EDX). The extinction spectra were measured with a UV-VIS-NIR spectrophotometer (Varian Cary 5000). The SERS spectra were measured with a high-resolution confocal Raman microscope (Horiba Jobin-Yvon Lab Ram HR) under the excitation of 488 nm and 785 nm diode lasers. The laser beam was focused onto the sample through a × 50 N.A. 0.75 objective lens. The focus spot areas of the 488 nm and 785 nm lasers on the sample were approximately 3.2 × 10^–6^ mm^2^ and 1.76 × 10^–6^ mm^2^, respectively. Signal acquisition time was 3 s. The time-dependent absorption spectra of the reaction solution were measured using a UV-vis spectrophotometer (TU-1810).

## Results and Discussion

**Fig. 1:**
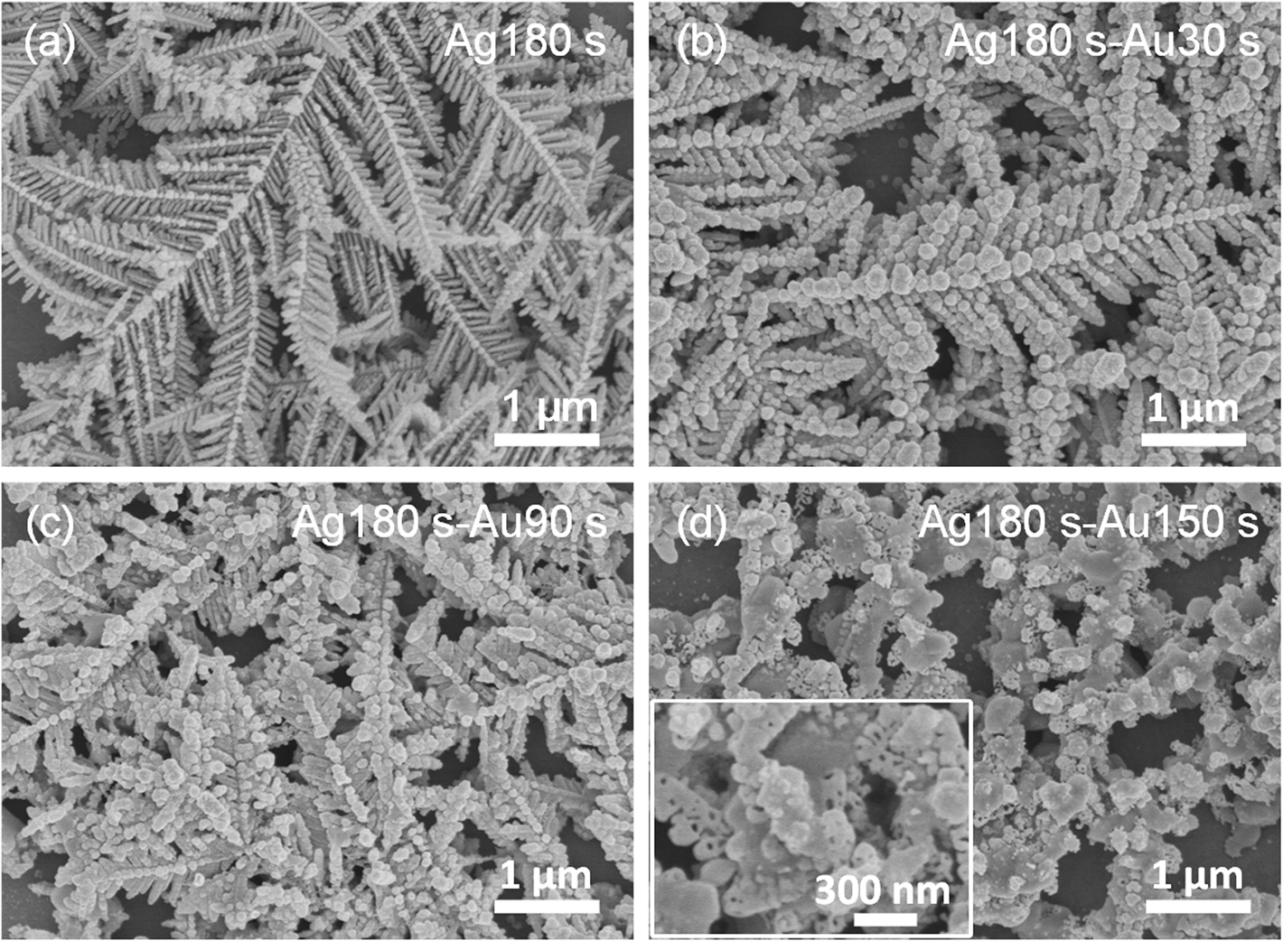
SEM images of the **a** dendritic Ag nanostructures and **b**–**d** dendritic Au/Ag bimetallic nanostructures prepared after different replacement reaction time: 30, 90, and 150 s, respectively. For simplicity, these samples were designated as bimetallic nanostructure (Ag180s-Au0s), (Ag180s-Au30s), (Ag180s-Au90s), and (Ag180s-Au150s), respectively. Inset is the corresponding high-magnification SEM image

Figure 1a shows the SEM image of initial dendritic Ag nanostructures prepared by electrochemical deposition for 180 s. The image clearly shows that the dendritic Ag nanostructure has a hierarchical fractal structure with a large surface area, abundant branches, tips, edges, and nanogaps. The electrochemical deposition of dendritic Ag nanostructures is a non-equilibrium growth process. The growth mechanism can be interpreted with a diffusion-limited aggregation model [31]. Figure 1b–d shos the morphological and structural changes of the samples during the replacement reaction for different time (30, 90, and 150 s) in the HAuCl_4_ solution. After short reaction time (*t* < 90 s), the whole structure of the sample was still the initial dendritic nanostructure (Fig. 1)b, c. During the replacement reaction, the oxidation of Ag atoms (Ag^0^) into Ag ions (Ag^+^) led to the gradual consumption of Ag dendrites and Au ions (Au^3+^) were simultaneously reduced to Au atoms (Au^0^) on the surface of Ag dendrites. The Au atoms deposited on the surface of dendritic Ag nanostructures formed a large number of Au nanoparticles and the initial edgy branches quickly evolved into more rod or spherical shapes, thus resulting in a smaller gap. However, after longer replacement reaction time (150 s), the dendritic structure was broken to form leaf-like rods and particles and a large number of pores and cavities appeared due to the removal of Ag from the initial Ag dendrite (Fig. 1d).

**Fig. 2:**
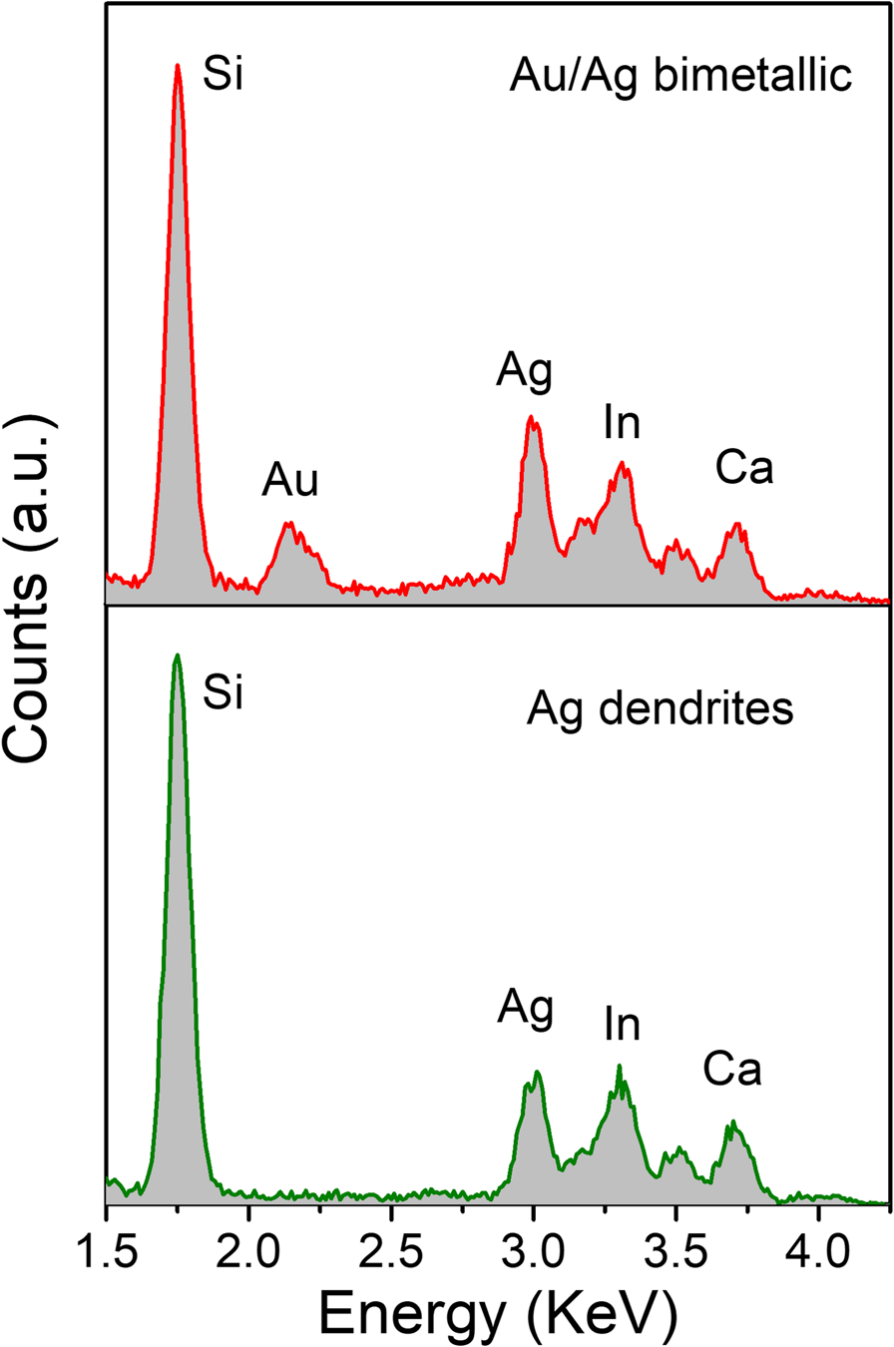
EDX spectra of the dendritic Ag nanostructures (Ag180s-Au0s) and dendritic Au/Ag bimetallic nanostructures (Ag180s-Au30s)

To further examine the composition of the nanostructures, EDX measurements were performed (Fig. 2). In the EDX spectra of the initial Ag dendrite, in addition to the characteristic peaks generated by the ITO glass, only the Ag characteristic peak was observed. The Au characteristic peak also appeared in the EDX spectrum of the samples prepared by replacement reaction in HAuCl_4_ for 30 s, confirming that the dendritic nanostructures prepared by the displacement reaction were Au/Ag bimetallic nanostructures.

**Fig. 3:**
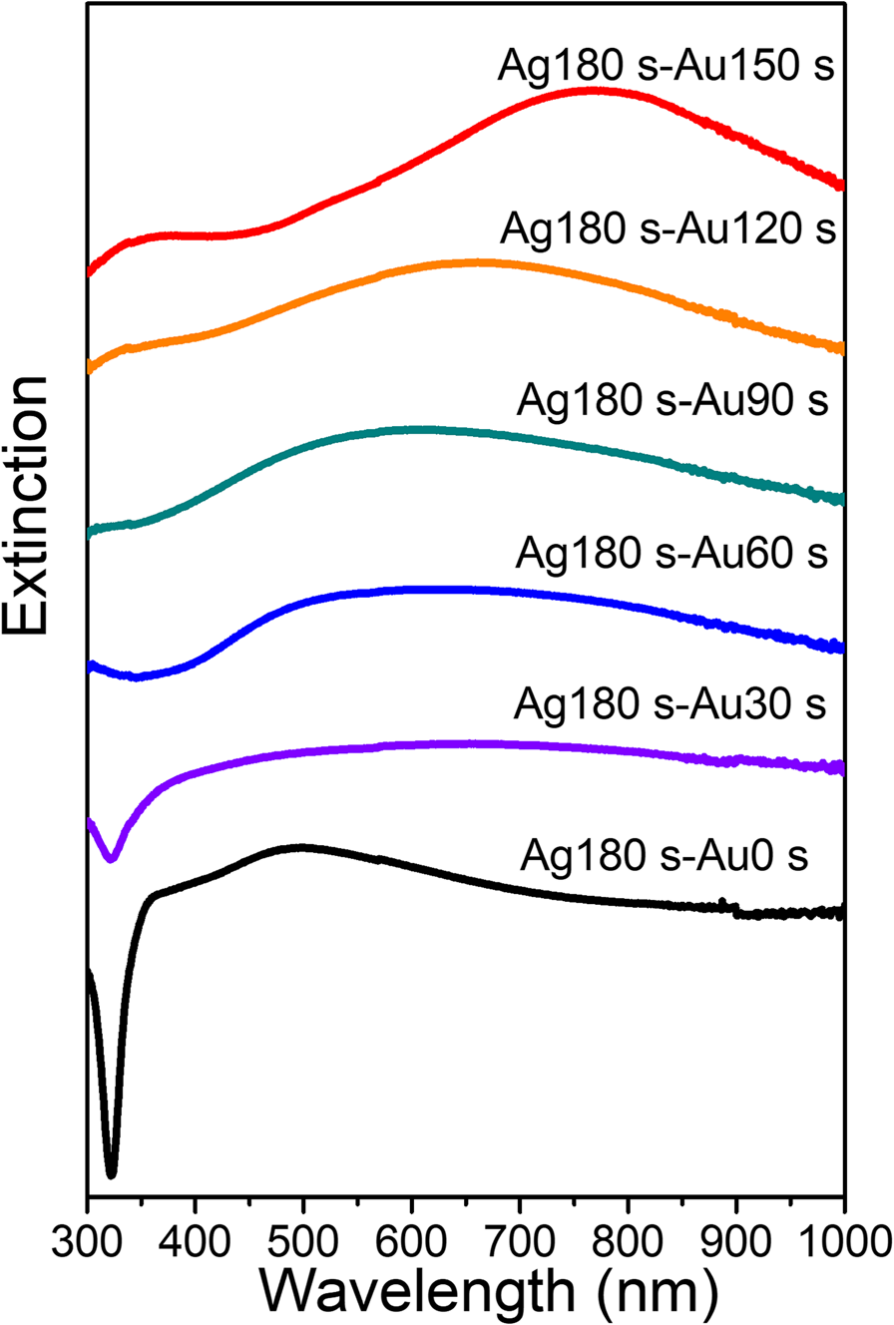
Extinction spectra of the dendritic Ag nanostructures and dendritic Au/Ag bimetallic nanostructures. The spectra are stacked from bottom to top with the increase in replacement reaction time

The plasmon properties of dendritic Au/Ag bimetallic nanostructures were systematically investigated by measuring the extinction spectra of the dendritic nanostructures prepared at different replacement time (Fig. 3). The initial dendritic Ag nanostructures show a broad plasmon resonance with a peak around at 490 nm. The dendritic Au/Ag bimetallic nanostructures (Ag180s-Au30s) exhibited a broader plasmon resonance than the initial dendritic Ag nanostructures due to the plasmon resonance of the Au nanoparticles (comparable resonance strength of Ag dendrites and Au nanoparticles). As replacement reaction time increased, the plasmon resonance peaks of the dendritic Au/Ag bimetallic nanostructures gradually red-shifted and were narrowed due to the increased resonance strength of Au nanoparticles (caused by the accumulation of Au nanoparticles and gradual consumption of Ag dendrites). In the extinction spectra of bimetallic nanostructures (Ag180s-Au150s), two resonance peaks around 775 nm and 362 nm were observed due to the transformation of dendritic nanostructures into leaf-like nanorods and nanoparticles.

**Fig. 4:**
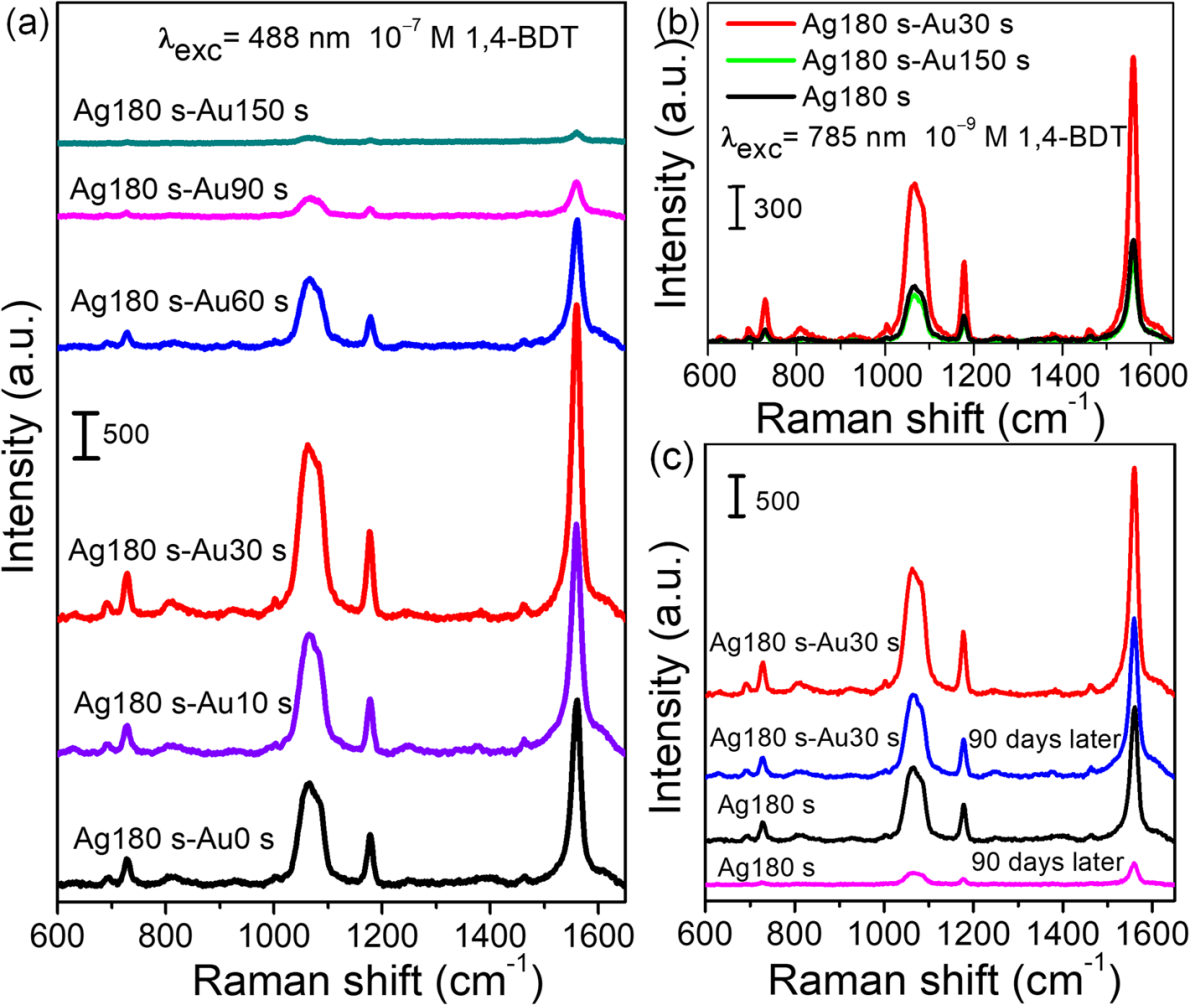
**a**, **b** SERS spectra of 10^−9^ M 1,4-BDT adsorbed on dendritic Ag nanostructures and Au/Ag bimetallic nanostructures excited at 488 nm and 785 nm, respectively. **c** SERS spectra of 10^−9^ M 1,4-BDT detected at the freshly prepared and 90-day-old substrates, respectively. Curves are shifted vertically for clear presentation

Figure 4a shows the SERS spectra of 1,4-BDT (10^−9^ M) molecules adsorbed on the dendritic nanostructures measured at an excitation wavelength of 488 nm. In the SERS spectra, four main peaks at 730, 1067, 1178, and 1563 cm^−1^ were consistent with previous reports for 1,4-BDT [32, 33]. When replacement reaction time increased to 30 s, the SERS intensities of dendritic Au/Ag bimetallic nanostructures gradually increased and were stronger than that of dendritic Ag nanostructures. However, the SERS intensity decreased sharply when reaction time increased from 30 s to 150 s, indicating the importance of replacement reaction time in the optimization of the SERS enhancement of dendritic Au/Ag bimetallic nanostructures. For short replacement reaction time (*t* < 30 s), the gap between the branches was reduced due to the formation of a large number of small-sized Au nanoparticles on the surface of Ag dendrites, thus resulting in a stronger local field enhancement confined in the inter-branch gaps [15]. Therefore, the SERS intensity of dendritic Au/Ag bimetallic nanostructures was greatly enhanced compared to that of dendritic Ag nanostructures. After reaching the maximum value, the SERS intensity decreased sharply with the increase in replacement reaction time for the following reasons. Firstly, the increased number of Au nanoparticles on the surface of Ag dendrites and the SERS enhancement were mainly derived from Au rather than the Ag with a larger SERS enhancement factor [14, 16]. Secondly, the breakdown of dendritic nanostructure resulted in the disappearance of a large number of SERS hotspots [11]. Thirdly, the plasmon resonance red-shifted toward the excitation wavelength. The excitation wavelength at 785 nm was closer to the plasmon resonance wavelength of the bimetallic nanostructure prepared after long replacement time, whereas the SERS intensity of dendritic Au/Ag bimetallic nanostructure (Ag180s-Au30s) was still stronger than that of the bimetallic nanostructure (Ag180s-Au150s) (Fig. 4b). The difference strongly suggested that the morphology was mainly responsible for the significant SERS enhancement of bimetallic nanostructures with replacement reaction time *t* > 30 s.

The SERS enhancement factor (EF) is calculated with EF = (*I*_SERS_ × *N*_Raman_)/(*I*_Raman_ × *N*_SERS_) to compare the signal intensity of the peak at 1563 cm^−1^, where *I*_SERS_ and *I*_Raman_ are the signal intensities for 1,4-BDT molecules adsorbed on the surface of dendritic Au/Ag bimetallic nanostructure (Ag180s-Au30s) and a glass plate (normal Raman measurement), respectively. *N*_SERS_ and *N*_Raman_ are the numbers of molecules for SERS measurement and normal Raman measurement, respectively. Herein, 50 μL of 10^−9^ M and 10^−2^ M 1,4-BDT ethanol solutions were dispersed on the dendritic Au/Ag bimetal nanostructure substrate and glass substrate with the same area (25 mm^2^), respectively. We assumed that the 1,4-BDT molecules were uniformly dispersed on the substrate and that all the molecules within the laser spot were illuminated and contributed to the SERS and Raman spectra. Under the excitation at 488 nm, the SERS enhancement factor of dendritic Au/Ag bimetallic nanostructure (Ag180s-Au30s) is calculated to be 6.1 × 10^8^, which is much larger than those of Ag@Au concave cuboctahedra (4.8 × 10^6^) [20], hybrid Au−Ag nanochains (2.4 × 10^7^) [34], double-shelled Au/Ag nanoboxes (6.6 × 10^5^) [35], and flower-like 3D Ag-Au heteronanostructures (1.17 × 10^7^) [36].

The time stability of as-prepared SERS substrates is significant for their applications. It had been extensively reported that Au/Ag bimetallic nanostructures exhibited the better SERS activities and time stability than Ag-based SERS substrates [13–18]. In this work, we also evaluated the time stability of the dendritic Ag and Au/Ag bimetallic nanostructure substrates (Fig. 4c). The SERS signal intensity of the Ag dendrite substrate was decreased by ~ 84% after 90 days due to the oxidation under ambient conditions. In contrast, the SERS intensity of 1,4-BDT adsorbed on dendritic Au/Ag bimetallic nanostructures (Ag180s-Au30s) was only decreased by ~ 30% after 90 days, indicating that the dendritic Au/Ag bimetallic nanostructure substrate had the long-term stability. Dendritic Ag nanostructures had been reported to exhibit super-SERS sensitivity and ultra-high electromagnetic enhancement factor [24]. Our previous study also confirmed that the Ag dendrite fractal nanostructures exhibited more significant SERS enhancement and achieved a low detection limit of 10^−14^ M 1,4-BDT [22]. As mentioned above, dendritic Au/Ag bimetallic nanostructures exhibited the better SERS enhancement effect and long-term stability than the dendritic Ag nanostructures and were more suitable SERS platforms.

**Fig. 5:**
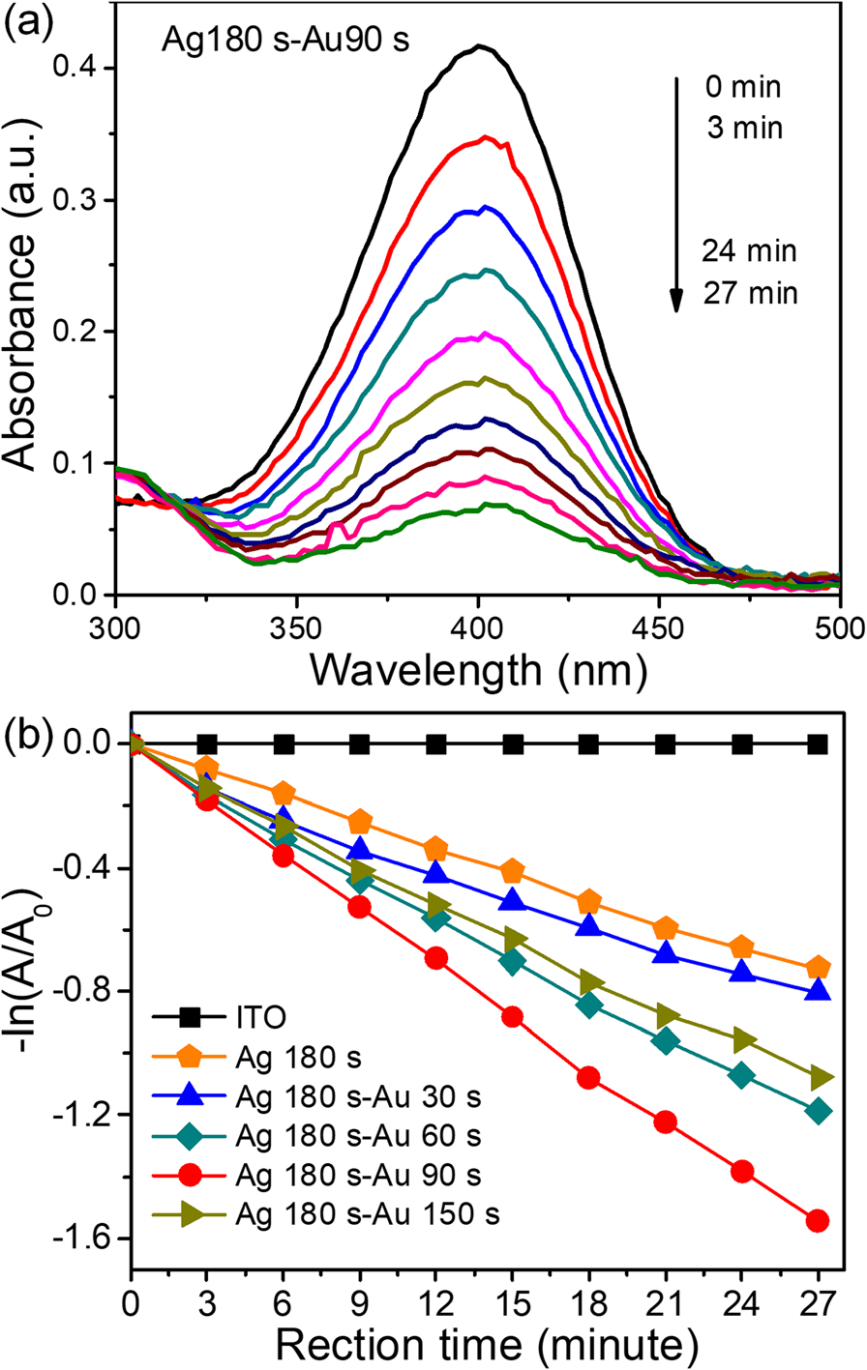
**a** Time-dependent UV-vis absorption spectra of the reduction of 4-NP by NaBH_4_ in the presence of dendritic Au/Ag bimetallic nanostructures (Ag180s-Au90s). **b** Plots of -ln(A/A_0_) at *λ* = 400 nm as a function of reaction time in the presence of catalysts with the same area

Dendritic metal nanostructures possess a large surface area, multiple branches, tips, and edges with low coordination numbers and provide a large number of highly active sites for breaking chemical bonds [27–29]. In this work, the catalytic activities of the dendritic nanostructures were evaluated by the model reaction of the catalytic reduction of 4-NP by NaBH_4_ to 4-aminophenol (4-AP). In the time-dependent absorption spectra of the reaction solution in the presence of dendritic Au/Ag bimetallic nanostructures (Ag180s-Au90s), the intensity of the absorption peak at 400 nm gradually decreased and a new absorption peak at 300 nm corresponding to 4-AP was observed (Fig. 5a) [9–11]. The results indicated that the dendritic Au/Ag bimetallic nanostructures were efficient catalysts for this reduction reaction. Fig. 5b shows the plots of -ln(A/A_0_) at 400 nm as a function of reaction time in the presence of catalysts with the same area. The estimated values of the rate constant *k* were 0 min^−1^ (ITO glass), 2.68 × 10^−2^ min^−1^ (Ag180s-Au0s), 2.91 × 10^−2^ min^−1^ (Ag180s-Au30s), 4.37 × 10^−2^ min^−1^ (Ag180s-Au60s), 5.76 × 10^−2^ min^−1^ (Ag180s-Au90s), and 3.95 × 10^−2^ min^−1^ (Ag180s-Au150s), respectively. The effect of ITO glass on catalysis was negligible. The reaction rate gradually increased when replacement reaction time increased from 0 to 90 s and decreased when replacement reaction time was 150 s. The dendritic Au/Ag bimetallic nanostructures (Ag180s-Au90s) exhibited the highest reaction rate, which was ~ 2 times higher than that of the dendritic Ag nanostructures. The significant enhancement in the catalytic activity of dendritic Au/Ag bimetallic nanostructures (Ag180s-Au90s) could be attributed to the synergistic effects between the two metals and provided more intermetallic interfaces, where electronic structures were changed [4, 5, 11]. As the Fermi level for Au (− 5.0 eV) was lower than that for Ag (−4.6 eV), the charge transfer from Ag to Au led to the formation of an electron-enriched region in Au near the Au/Ag interface [11, 37]. The presence of these surplus electrons facilitated the degradation of 4-NP molecules near these regions. The more interfaces there are, the more chances for 4-NP molecules to be adsorbed in such regions with surplus electrons, thus leading to a higher catalytic rate. After reaching the maximum value, the reaction rate decreased with the increase in replacement reaction time. The decrease might be interpreted as follows. Firstly, as replacement reaction time increased from 90 s, Au nanoparticle shell covered Au/Ag interfaces and Ag was further depleted, so the number of accessible interfaces decreased again. Secondly, the breakdown of dendritic nanostructure resulted in a decrease in the number of catalyst active sites. The reaction rate of Au/Ag bimetallic nanostructures (Ag180s-Au150s) was higher than that of dendritic Au/Ag bimetallic nanostructures (Ag180s-Au30s) due to the large number of pores and cavities in the bimetallic nanostructures (the inset in Fig. 1d).

## Conclusion

In the study, we fabricated a bifunctional dendritic Au/Ag bimetallic nanostructure by combining the electrochemical deposition and replacement reaction. A tunable localized surface plasmon resonance (LSPR), SERS and catalytic activity were obtained by tuning replacement reaction time (morphology and composition). Experimental results demonstrated that the fabricated dendritic Au/Ag bimetallic nanostructure (Ag180s-Au30s) substrates exhibited the better SERS activity and prominent long-term stability due to the formation of Au nanoparticle shell on the surface of Ag dendrites. The catalytic activity of dendritic Au/Ag bimetallic nanostructures (Ag180s-Au90s) for the catalytic reduction of 4-NP by NaBH_4_ was enhanced by two times compared to that of the initial dendritic Ag nanostructures. These experimental results indicated that the dendritic Au/Ag bimetallic nanostructures could serve as bifunctional substrates with both SERS and catalytic activity for the potential applications in in-situ SERS monitoring of catalytic reactions [20, 21].

## Data Availability

All data generated or analysed during this study are included in this published article.

## Abbreviations

SERS: Surface-enhanced Raman scattering
4-NP: 4-nitrophenol
LSPR: Localized surface plasmon resonance
ITO: Indium tin oxide
SEM: Scanning electron microscope
EDX: Energy-dispersive X-ray spectroscopy
1,4-BDT: 1,4-benzenedithiol
